# Diagnostic Time Lag of Pediatric Haemophagocytic Lymphohistiocytosis and Patient Characteristics: A Retrospective Cohort Study

**DOI:** 10.3389/fped.2021.692849

**Published:** 2021-06-17

**Authors:** Xun Li, Haipeng Yan, Zhenghui Xiao, Xinping Zhang, Jiaotian Huang, Shi-Ting Xiang, Mincui Zheng, Zhenya Yao, Ping Zang, Desheng Zhu, Liping Li, Xiulan Lu

**Affiliations:** ^1^Pediatrics Research Institute of Hunan Province, Hunan Children's Hospital, Changsha, China; ^2^Department of Pediatric Intensive Care Unit, Hunan Children's Hospital, Changsha, China; ^3^Department of Pediatric Hematology, Hunan Children's Hospital, Changsha, China

**Keywords:** haemophagocytic lymphohistiocytosis, haemophagocytosis, diagnostic criteria, risk factor, time lag

## Abstract

The difficulties and challenges of applying the HLH-2004 diagnostic criteria to early identification and diagnosis of haemophagocytic lymphohistiocytosis have been fully addressed in previous studies. However, the distribution of the diagnostic time lag of haemophagocytic lymphohistiocytosis and related patient characteristics remain unclear. This study investigated the time lags between symptom onset and diagnosis and between hospital admission and diagnosis among pediatric patients with haemophagocytic lymphohistiocytosis, and identified factors that associated with a shorter or longer diagnostic time lag. The cohort of patients with haemophagocytic lymphohistiocytosis was drawn from a tertiary children's hospital and consisted of 122 pediatric patients. The distributions of symptom-to-diagnosis and admission-to-diagnosis time lags were assessed. Clinical characteristics within 48 h of admission and the fulfillment of HLH-2004 diagnostic criteria were compared among admission-to-diagnosis time lag categories. Logistic regression analyses were conducted to identify factors associated with an admission-to-diagnosis time lag >3 days. The median interval from first symptom onset to HLH diagnosis was 12 days (range 4–71 days) and the median interval from hospital admission to HLH diagnosis was 2 days (range 0–23 days). The following factors were negatively associated with admission-to-diagnosis > 3 days: Epstein–Barr virus infection; admission through pediatric intensive care unit; diagnosis established without NK-cell activity and soluble CD25 tests; the performance of all readily available diagnostic tests for HLH (within 48 and 72 h); concurrent fever, splenomegaly, and cytopenias within 48 h; hemophagocytosis, hypertriglyceridemia and/or hypofibrinogenemia within 48 h; and elevated ferritin, total bilirubin, alanine aminotransferase, and prothrombin time within 48 h. Our findings suggest that performance of adequate diagnostic tests for HLH is essential for early diagnosis of HLH. Once suspected, immediate and adequate diagnostic tests for HLH should be arranged for PICU patients. Improvements in diagnostic procedures and monitoring plans are needed to promote early diagnosis of HLH.

## Introduction

Haemophagocytic lymphohistiocytosis (HLH) is a disorder characterized by extreme immune activation, which results in hypercytokinaemia and immune-mediated injuries to multiple organ systems (1–3). HLH is classified as primary or secondary (1). Primary HLH, also known as familial HLH or genetic HLH, demonstrates clear familial inheritance or genetic causes. Secondary HLH occurs in patients without a family history or a genetic cause, and these patients typically have an underlying disease that triggers the HLH, such as infection, malignancy, and/or an autoimmune disorder. HLH secondary to a rheumatologic or auto-inflammatory disease is also referred to as macrophage activation syndrome (MAS) (4, 5). Notably, patients with susceptibility genes for HLH can experience disease onset as a result of concurrent infection or another medical condition. Patients with HLH who have a genetic cause but lack adequate genetic tests might be clinically classified as a secondary HLH. Importantly, both primary and secondary types of HLH are life-threatening (1). The diagnostic criteria and therapeutic guidelines for HLH published by the Histiocyte Society in 1994 and 2004 (HLH-94 and HLH-2004, respectively) substantially improved the survival of patients with HLH (6, 7). Nonetheless, HLH-related early mortality remains high, with reported 30-day overall survival (OS) rates ranges from 70 to 80% among pediatric patients (8–11). For pediatric patients with either primary or secondary HLH, delayed initiation of HLH treatment is a risk factor for early death, therefore timely diagnosis and treatment are essential for survival (3, 12, 13).

However, initiating timely HLH treatment is hindered by the challenge of rapidly establishing the diagnosis. Given the non-specific clinical presentation of HLH and the overlap between its symptoms and those of other inflammatory diseases, such as sepsis, the diagnosis of HLH is often delayed (12, 14). Moreover, HLH can rapidly progress to multiple organ dysfunction syndrome (MODS) or even death (15, 16), such that the time frame for an early diagnosis allowing effective treatment is short. According to the HLH-2004 criteria, a molecular diagnosis or at least five of the eight criteria should be met for the establishment of the HLH diagnosis (1). However, with regard to the clinical course, these criteria are often not fulfilled at presentation (17), and waiting for their appearance to confirm HLH could delay treatment (3). A further complication is that two of the criteria consist of natural killer cell (NK-cell) activity and soluble CD25 (sCD25) measurement, respectively, although these tests are often not part of routine practice (18). Efforts have been made to search for new diagnostic markers of HLH, and several promising candidate markers have been proposed, including soluble CD163, interferon-γ, and interleukin (IL)-10 (19, 20). However, their better performance compared with existing diagnostic criteria in achieving an early diagnosis awaits demonstration. Their success would benefit especially those HLH patients whose diagnosis is missed or delayed based on the potential ambiguity of the current diagnostic criteria.

Analysis of the literature concerning delayed diagnosis of HLH indicates that both the scope of the problem and the characteristics of affected patients are unclear (3, 12, 21). However, insights into the numbers and characteristics of patients with a delayed HLH diagnosis could contribute to the development of early diagnosis strategies, and may help to identify a subset of patients who require careful consideration in the search for effective diagnostic markers. This study investigated two types of time lag in the diagnosis of HLH: admission-to-diagnosis and symptom-to-diagnosis. The admission-to-diagnosis time lag was the main study variable, which was defined as the interval from hospital admission to the day of confirmed HLH diagnosis. The admission-to-diagnosis time lag was used for the evaluation of the efficiency of diagnostic procedures and for the identification of where clinical improvements are needed. We also investigated patients' characteristics according to the interval from the first symptom onset to the day of confirmed HLH diagnosis (symptom-to-diagnosis time lag), as this time lag could indicate the duration of disease progression. Specifically, this study had three objectives. First, to investigate the distributions of symptom-to-diagnosis and admission-to-diagnosis time lags among HLH patients, and identify the associated patients' characteristics. Second, to investigate the degrees of fulfillment of the HLH-2004 diagnostic criteria among different admission-to-diagnosis time lag groups, and investigate how the rates of performing early diagnostic tests within 48 and 72 h affected the time lags. Third, to investigate the patients' clinical characteristics within 48 h of hospital admission and their association with diagnostic time lags. We hypothesized that patients with HLH who have a delayed diagnosis present to the hospital with fewer HLH-like characteristics.

## Materials and Methods

### Study Population

This was a retrospective cohort study that included pediatric patients (0 to 18 years of age) discharged (either alive or dead) from Hunan Children's Hospital in China with a diagnosis of HLH between June 2015 and October 2018. The chart review was conducted between May 2019 and November 2020. The survival status at day 30 after hospital admission was extracted from the medical record or followed up by a phone call. The exclusion criteria included patients diagnosed with HLH prior to the indicated hospital admission date; patients with essential diagnostic data missing; or patients > 18 years of age at the time of HLH diagnosis.

The study protocol was reviewed and approved by the Medical Ethics Committee of the Hunan Children's Hospital (HCHLL-2019-40) and have been performed in accordance with the ethical standards as laid down in the 1964 Declaration of Helsinki and its later amendments or comparable ethical standards. The requirement for written informed consent was waived by the Medical Ethics Committee of the Hunan Children's Hospital.

### Variables and Diagnostic Criteria

HLH was diagnosed according to the HLH-2004 criteria (1). According to HLH-2004 diagnostic criteria, the diagnosis of HLH can be established if one of either A or B below is fulfilled (1): A. A molecular diagnosis consistent with HLH. B. Five out of eight criteria fulfilled: (1) Fever. (2) Splenomegaly. (3) Cytopenias affecting 2 of 3 lineages in the peripheral blood. (4) Hypertriglyceridemia and/or hypofibrinogenemia: fasting triglycerides ≥3.0 mmol/L, fibrinogen ≤ 1.5 g/L. (5) Hemophagocytosis in bone marrow or spleen or lymph nodes. No evidence of malignancy. (6) Low or absent NK-cell activity. (7) Ferritin ≥ 500 μg/L. (8) sCD25 ≥ 2400 U/ml. Primary HLH was diagnosed based on a family history of HLH and/or a molecular (genetic) diagnosis of HLH. In the study center, the NK-cell activity and sCD25 tests were conducted by a third-party company (Wuhan Kindstar Diagnostics Co., Ltd). The genetic tests were conducted by multiple third-party companies. And the other tests were conducted in the study center.

In the study center, all hospitalized patients underwent assessments of sustained fever length, splenomegaly, and routine blood analysis on hospital admission. Triglycerides and fibrinogen levels were also routinely checked on admission in most patients, but not all patients, according to the clinical presentation and suspected diseases. Ferritin and haemophagocytosis assessments were performed if HLH or other related diseases were suspected. The HLH diagnostic procedure in the study center was that if a patient met more than three HLH criteria in routine assessments, diagnostic tests were performed to determine HLH susceptibility. Initial evaluation typically took up to 48 h and most amended tests results (except for NK-cell activity, sCD25, and genetic tests) would be received within 72 h of admission. During the study period, NK-cell activity and sCD25 tests were not routine and were performed only if a patient demonstrated robust signs of HLH (or other relevant diseases), and results typically were not received within 72 h. Genetic test was performed in only a few patients due to high cost and long result turnaround time. Patients who have suspected HLH but do not fulfill the diagnostic criteria would be further monitored by repeating the diagnostic tests for HLH, however the test interval was determined based on the clinical presentation and was not standardized in the study center.

The main study variable was the admission-to-diagnosis time lags. The admission-to-diagnosis time lag was defined as the interval (in days) between hospital admission and the day of confirmed HLH diagnosis. Because the diagnostic procedure for initial evaluation and amended tests for HLH could require up to 3 days, patients were categorized into two admission-to-diagnosis time lag groups: ≤ 3 days and > 3 days. Presumably, following the normal diagnosis procedure and using current diagnostic criteria, most patients with suspected HLH should have received a diagnosis in ≤3 days of hospitalization. Patients diagnosed after 3 days were likely to have progressed from subclinical HLH to HLH or had a delayed diagnosis. The patients' characteristics according to symptom-to-diagnosis time lag categories were also investigated. The symptom-to-diagnosis time lag was defined as the interval (in days) from the reported first symptom onset to the day of confirmed HLH diagnosis. Patients were categorized into two groups based on the median value of symptom-to-diagnosis time lag: ≤12 days and > 12 days.

Because fever, splenomegaly, and cytopenias were routinely checked in general clinical practice and their fulfillment would raise susceptibility to HLH, we investigated whether the concurrent fulfillment of these three items was associated with a shorter diagnostic time lag. Tests of NK-cell activity and sCD25 are not immediately available in many clinical settings, which may limit the application of the HLH-2004 criteria. To determine how these two criteria affected the diagnostic procedure, we investigated the numbers of patients who could establish an HLH diagnosis without NK-cell activity and sCD25 tests, then analyzed their associations with diagnostic time lags. To investigate the performance of early diagnostic procedures among patients according to diagnostic time lag categories, we calculated the proportions of patients who received all readily available tests for HLH (all items except for NK-cell activity, sCD25, and genetic tests) within 48 and 72 h of hospital admission.

Other investigated variables were: general characteristics, including age, sex, hospital admission department; survival status at day 30 after hospital admission; underlying diseases/potential triggers of HLH, including primary HLH, autoimmune disorders, malignancy, sepsis [diagnosed as described in (22)], infections, and Epstein-Barr virus (EBV) infection (including acute and chronic active infection, tested by VCA-IgM, VCA-IgG, EBNA-IgG, and/or EBV-DNA); concomitant diagnoses, including myocardial damage, heart failure (23), shock (24, 25), central nervous system disease (CNS) disease, hepatic dysfunction (26), respiratory failure, acute respiratory distress syndrome (ARDS) (27), severe pneumonia (28), coagulopathy, disseminated intravascular coagulation (DIC), acute kidney injury (29), gastrointestinal disorder, and MODS. Patients with HLH were treated using HLH-94/HLH-2004 protocol (1, 30). HLH-related treatment, including dexamethasone, etoposide, cyclosporine A, and intrathecal injection, were categorized into no use, used before diagnose, and used after diagnose groups.

Concomitant diagnosis, symptoms, and laboratory tests within 48 of admission were investigated, including all HLH-2004 diagnostic items except for sCD25 and genetic tests (none of the results was reported within 48 h), diagnoses, and other common lab tests like total bilirubin (TBil), alanine aminotransferase (ALT), prothrombin time (PT), activated partial thromboplastin time (APTT), and INR.

### Statistical Analysis

Categorical variables were presented as absolute values and percentages. Continuous variables are presented as mean (standard deviation), or median, range and quartiles (Q1 and Q3), as appropriate. Between-group comparisons for categorical variables were conducted using chi-squared test or Fisher's exact test, as appropriate. Between-group comparisons for continuous variables were conducted by Wilcoxon rank sum test. Logistic regression analysis were conducted to assess the factors associated with an admission-to-diagnosis time lag >3 days. The correlation between the symptom-to-diagnosis time lag and the admission-to-diagnosis time lag was tested by Spearman correlation analysis. All tests were set two-tailed with a type 1 error rate fixed at 5%. Missing data was not imputed. All statistical analyses were performed using SAS 9.3 (SAS Institute, Inc., Cary, NC).

### Additional Tests and Sensitivity Analysis

Additional tests were conducted to explore the diagnostic characteristic according to factors which were found to be associated with a shorter or longer diagnostic time lag. Two sensitivity analyses were conducted to determine whether the results of between-group comparisons for 30-day OS rates would change if different cut-off points were used for the time lag categorization.

## Results

**Figure 1:**
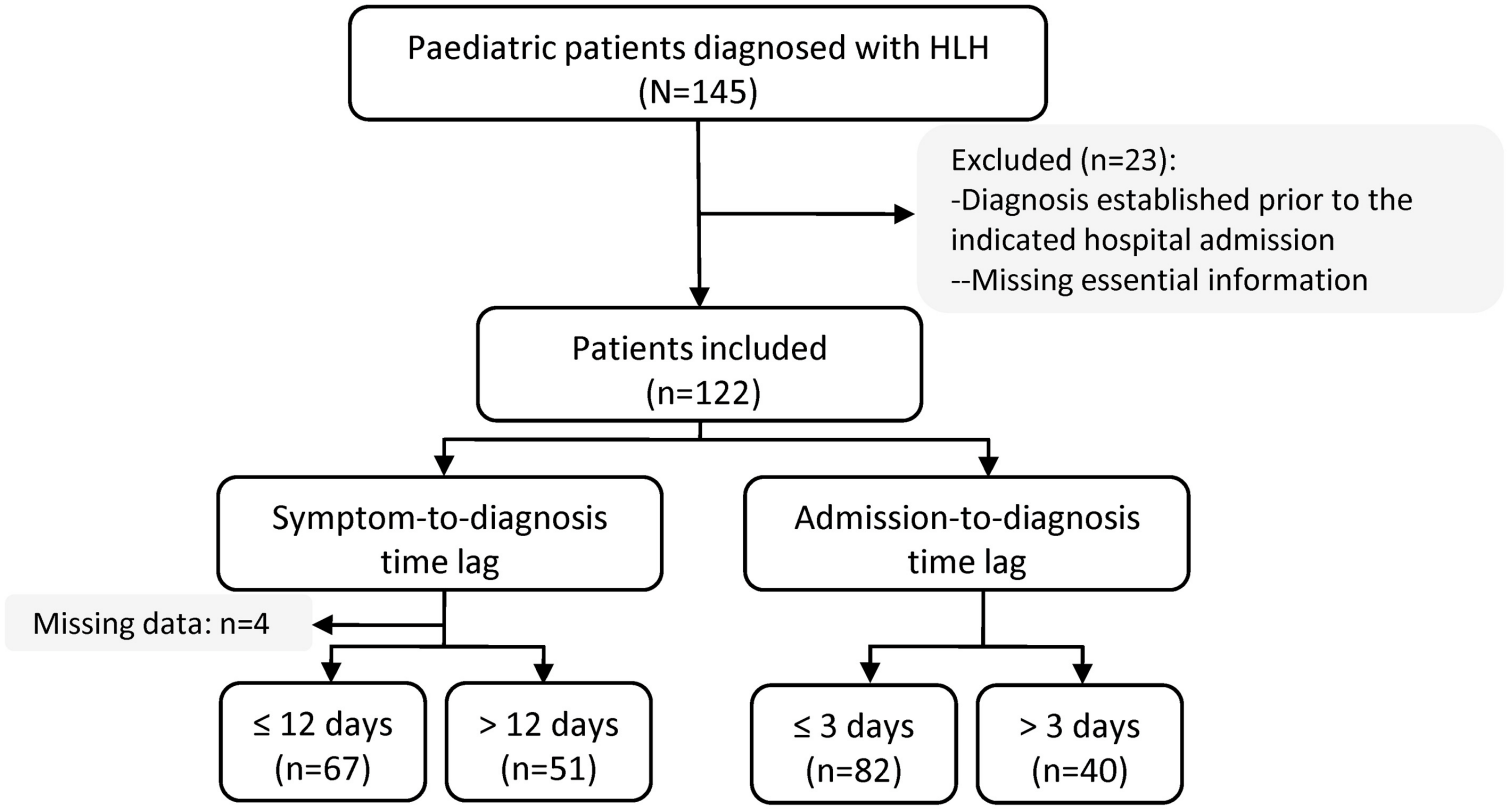
Flow diagram of study population. HLH, haemophagocytic lymphohistiocytosis.

**Figure 2:**
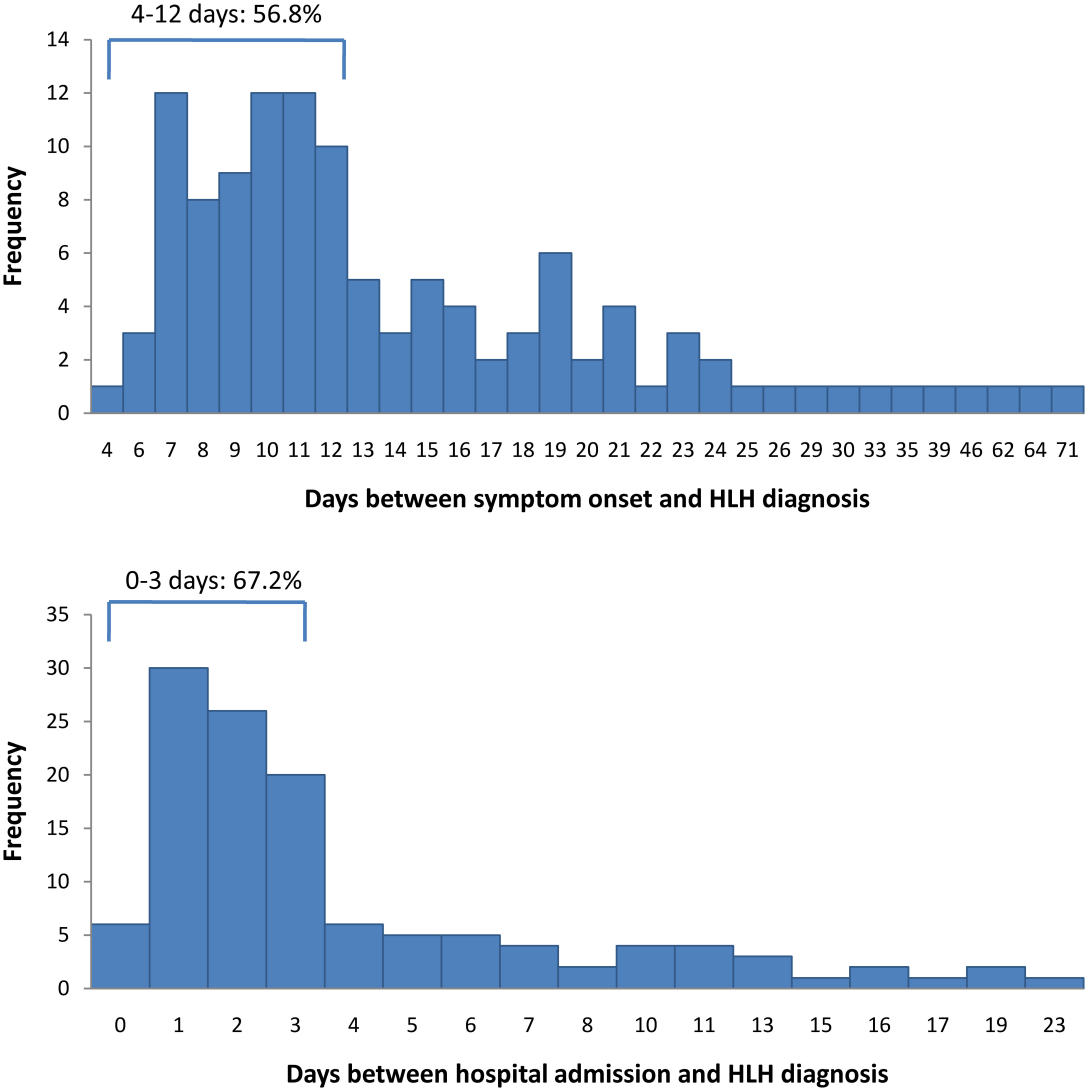
Distribution of diagnostic time-lags among 122 patients with haemophagocytic lymphohistiocytosis. Top: first symptom onset to diagnosis time lag. Bottom: hospital admission to diagnosis time lag. HLH, haemophagocytic lymphohistiocytosis.

Between June 2015 and October 2018, 145 patients were discharged with a diagnosis of HLH. Of these, 23 patients were excluded from this study, either because they had been diagnosed with HLH prior to the indicated hospital admission date or because essential information was missing, leaving 122 patients for the analysis (Figure 1). Four patients had missing data concerning the symptom-to-diagnosis time lag. For the remaining 118 patients, the median time lag between first symptom onset and HLH diagnosis was 12 days (Q1–Q3: 9–18 days). The median time lag between hospital admission and HLH diagnosis was 2 days (Q1–Q3: 1–5 days). In total, 82 patients (67.2%) were diagnosed with HLH within 72 h after hospital admission, and 40 (32.8%) were diagnosed later. Figure 2 presents distribution of diagnostic intervals among the 122 patients. Longer symptom-to-diagnosis time lags were significantly associated with longer admission-to-diagnosis time lags (Spearman correlation coefficient = 0.416, *p* < 0.0001; also see Table 1).

**Table 1:** General characteristics of 122 pediatric HLH patients according to HLH diagnostic time lag categories.

	**All**	**First symptom to diagnosis^†^**			**Hospital admission to diagnosis**		
		**≤12 days**	**>12 days**	***P***	**≤3 days**	**>3 days**	***P***
Total	122	67	51		82	40	
**Sex**, ***n*** **(%)**							
Male	68 (55.7)	36 (53.7)	30 (58.8)	0.7084	44 (53.7)	24 (60.0)	0.5080
Female	54 (44.3)	31 (46.3)	21 (41.2)		38 (46.3)	16 (40.0)	
**Age**, ***n*** **(%)**							
<1 year	18 (14.8)	8 (11.9)	10 (19.6)	0.6392	8 (9.8)	**10 (25.0)**	**0.0081**
≥1 year to 4 years	80 (65.6)	44 (65.7)	33 (64.7)		56 (68.3)	24 (60.0)	
≥ 5 years to 9 years	15 (12.3)	9 (13.4)	5 (9.8)		14 (17.1)	**1 (2.5)**	
10 years to 15 years	9 (7.4)	6 (9.0)	3 (5.9)		4 (4.9)	**5 (12.5)**	
**Admission department**, ***n*** **(%)**							
PICU	52 (42.6)	33 (49.3)	18 (35.3)	0.1389	**41 (50.0)**	**11 (27.5)**	**0.0183**
Other departments	70 (57.4)	34 (50.7)	33 (64.7)		**41 (50.0)**	**29 (72.5)**	
**First symptom to hospital admission (days)**							
Median (Q1,Q3)	**8 (6, 11)**	**7 (5, 9)**	**13 (9, 20)**	** <0.0001**	9 (7, 12)	7 (5, 10.5)	0.1594
Min, max	**2, 60**	**3, 12**	**2, 60**		3, 60	2, 60	
**Time gap between first symptom and HLH diagnose**, ***n*** **(%)**							
≤ 12 days	67 (56.8)	67 (100)	0		**55 (70.5)**	**12 (30.0)**	** <0.0001**
>12 days	51 (43.2)	0	51 (100)		**23 (29.5)**	**28 (70.0)**	
**30-day outcome**^**^‡^**^, ***n*** **(%)**							
Survive	82 (68.9)	42 (63.6)	36 (73.5)	0.3154	52 (65.0)	30 (76.9)	0.2117
Non-survive	37 (31.1)	24 (36.4)	13 (26.5)		28 (35.0)	9 (23.1)	

*HLH, haemophagocytic lymphohistiocytosis; PICU, pediatric intensive care unit; Q1, the first quartile; Q3, the third quartile*.

*Values in bold are statistically significant (p <0.05)*.

† *Four patients had missing data for the symptom-to-diagnosis time lag*.

‡ *Three patients were lost to follow-up at day 30*.

Table 1 lists the general characteristics of the patients and the 30-day OS rates, stratified according to HLH diagnostic time lag categories. Patients were aged from 1 month to 15 years old. Among the 122 patients, 42.6% were directly admitted to the pediatric intensive care unit (PICU) at hospital admission. The interval from first symptom onset to hospital admission was shorter among patients with a symptom-to-diagnosis time lag ≤12 days (compared with the >12 days group, *p* < 0.0001). Three patients were lost to follow-up at day 30. The 30-day OS rate for the study cohort was 68.9%. The differences in 30-day OS rates among time lag categories were not statistically significant (*p* > 0.05).

In this HLH cohort, nine patients (7.4%) were diagnosed with primary HLH; four patients (3.3%) had autoimmune disorders; six patients had malignancy (4.9%); 76 patients (62.3%) had concomitant sepsis; 113 patients (92.6%) had infection; and 83 patients (68.0%) had acute or chronic EBV infection. The EBV infection rate was significantly lower in the >3 days admission-to-diagnosis time lag group (45%) than in the ≤3 days group (79.3%, *p* = 0.0001). Differences in other underlying diseases and concomitant diagnosis among time lag groups were not statistically significant. Regarding the HLH-related treatment, one between-group difference was observed for the usage of dexamethasone: more patients in the admission-to-diagnosis time lag >3 days group were treated with dexamethasone before a diagnosis of HLH was established (≤3 days group: 13.4%, >3 days group: 30.0%; *p* = 0.0093).

Table 2 lists the fulfillment of the HLH-2004 diagnostic criteria during hospitalization, stratified according to diagnostic time lag categories. All 122 patients experienced cytopenias, as well as hypertriglyceridemia and/or hypofibrinogenemia. One patient with primary HLH only fulfilled four diagnostic items. Patients in the admission-to-diagnosis ≤3 days group fulfilled more diagnostic items (median = 7, Q1–Q3: 6–7), compared with patients in the >3 days group (median = 6, Q1–Q3: 5–7; *p* = 0.0023). The proportion of patients who met all three regularly checked criteria (fever, splenomegaly, and cytopenias) did not significantly differ among time lag categories (*p* > 0.05). More patients in the ≤3 days admission-to-diagnosis time group could have an HLH diagnosis established without NK-cell activity and sCD25 tests, compared with the corresponding number of patients in the >3 days group (91.5% and 77.5%, *p* = 0.0320).

**Table 2:** Fulfillment of the HLH-2004 diagnostic criteria during hospitalization according to HLH diagnostic time lag categories.

		**Hospital admission to diagnosis**		
	**All *n* (%)**	**≤3 days *n* (%)**	**> 3 days *n* (%)**	***P***
Total	122	82	40	
Fever	111 (91.0)	77 (93.9)	34 (85.0)	0.1744
Splenomegaly	112 (91.8)	75 (91.5)	37 (92.5)	1
Cytopenias	122 (100)	82 (100)	40 (100)	
Hemoglobin <90 g/L	118 (96.7)	80 (97.6)	38 (95.0)	0.5967
Platelets <100 × 10^9^ /L	115 (94.3)	78 (95.1)	37 (92.5)	0.6823
Neutrophils <1.0 × 10^9^/L	105 (86.1)	70 (85.4)	35 (87.5)	0.7493
Hypertriglyceridemia and/or hypofibrinogenemia	122 (100)	82 (100)	40 (100)	
Hypertriglyceridemia, ≥ 3.0 mmol/L	91 (74.6)	64 (78.0)	27 (67.5)	0.209
Hypofibrinogenemia, ≤ 1.5 g/L	104 (85.2)	**74 (90.2)**	**30 (75.0)**	**0.0258**
**Hemophagocytosis**				
No	24 (19.7)	17 (20.7)	7 (17.5)	1
Yes	89 (73.0)	62 (75.6)	27 (67.5)	
Not done	9 (7.4)	3 (3.7)	6 (15.0)	
**Low or absent NK-cell activity^†^**				
Yes	91 (74.6)	69 (84.1)	22 (55.0)	
Not done	31 (25.4)	13 (15.9)	18 (45.0)	
Ferritin ≥ 500 μg/L	112 (91.8)	**79 (96.3)**	**33 (82.5)**	**0.0140**
**Soluble CD25** **≥** **2400 U/ml^†^**				
No	3 (2.5)	3 (3.7)	0	
Yes	41 (33.6)	28 (34.1)	13 (32.5)	
Not done	78 (63.9)	51 (62.2)	27 (67.5)	
**No. of fulfilled items from 8 items**				
4 items	1 (0.8)	**0**	**1 (2.5)**	**0.0053**
5 items	19 (15.6)	**9 (11.0)**	**10 (25.0)**	
6 items	33 (27.0)	**21 (25.6)**	**12 (30.0)**	
7 items	49 (40.2)	**33 (40.2)**	**16 (40.0)**	
8 items	20 (16.4)	**19 (23.2)**	**1 (2.5)**	
Median (Q1,Q3)	7 (6, 7)	**7 (6, 7)**	**6 (5, 7)**	**0.0023**
Min, max	4, 8	**5, 8**	**4, 8**	
Fulfill three regularly checked criteria: fever, splenomegaly, and cytopenias	103 (84.4)	72 (87.8)	31 (77.5)	0.1406
Diagnosis established without NK-cell activity and soluble CD25 tests	106 (86.9)	**75 (91.5)**	**31 (77.5)**	**0.0320**

*HLH, haemophagocytic lymphohistiocytosis; NK-cell, natural killer cell*.

*Values in bold are statistically significant (p <0.05)*.

† *Between-group comparisons were not conducted due to missing data exceeds 30%*.

Table 3 lists symptoms and laboratory tests within 48 h of hospital admission. Compared with the >3 days admission-to-diagnosis time lag group, more patients in the ≤ 3 days group had splenomegaly, cytopenias, hypertriglyceridemia, hypofibrinogenemia, and hemophagocytosis, as well as elevated ferritin, TBil, ALT, APTT, PT, and INR within the first 48 h (all *p*-values <0.05). Patients in the ≤ 3 days group also fulfilled more HLH diagnostic items (median = 4, Q1–Q3: 4–5) within the first 48 h of hospital admission, compared with patients in the < 3 days group (median = 2, Q1–Q3: 2–3, *p* < 0.0001). The rates of performing all readily available HLH tests within 48 and 72 h were significantly higher in the admission-to-diagnosis ≤ 3 days group (*p* < 0.05). The results of between-group comparisons for concomitant diagnoses within 48 h are shown in Supplementary Table 1.

**Table 3:** Symptoms and laboratory tests within 48 h of hospital admission according to HLH diagnostic time lag categories.

		**Hospital admission to diagnosis**		
	**All *n* (%)**	**≤3 days *n* (%)**	**> 3 days *n* (%)**	***P***
Total	122	82	40	
**HLH-2004 criteria**^†^				
Fever	117 (95.9)	80 (97.6)	37 (92.5)	0.3292
Splenomegaly	89 (73.0)	**66 (80.5)**	**23 (57.5)**	**0.0073**
Cytopenias	82 (67.2)	**67 (81.7)**	**15 (37.5)**	** <0.0001**
Hemoglobin <90 g/L	73 (59.8)	**59 (72.0)**	**14 (35.0)**	**0.0001**
Platelets <100 × 10^9^ /L	94 (77.0)	**72 (87.8)**	**22 (55.0)**	**0.0001**
Neutrophils <1.0 × 1 0^9^ /L	65 (53.3)	**51 (62.2)**	**14 (35.0)**	**0.0047**
Hypertriglyceridemia and/or hypofibrinogenemia	76 (62.3)	**63 (76.8)**	**13 (32.5)**	** <0.0001**
**Hypertriglyceridemia**, **≥** **3.0 mmol/L**				
No	50 (41.0)	**33 (40.2)**	**17 (42.5)**	**0.0085**
Yes	41 (33.6)	**34 (41.5)**	**7 (17.5)**	
Not done	31 (25.4)	**15 (18.3)**	**16 (40.0)**	
**Hypofibrinogenemia**, **≤** **1.5 g/L**				
No	45 (36.9)	**29 (35.4)**	**16 (40.0)**	**0.0005**
Yes	64 (52.5)	**52 (63.4)**	**12 (30.0)**	
Not done	13 (10.7)	**1 (1.2)**	**12 (30.0)**	
**Hemophagocytosis**				
No	12 (9.8)	**7 (8.5)**	**5 (12.5)**	** <0.0001**
Yes	34 (27.9)	**33 (40.2)**	**1 (2.5)**	
Not done	76 (62.3)	**42 (51.2)**	**34 (85.0)**	
**Low or absent NK-cell activity^‡^**				
Yes	61 (50.0)	53 (64.6)	8 (20.0)	
Not done	61 (50.0)	29 (35.4)	32 (80.0)	
**Ferritin** **≥** **500** **μg/L**				
No	7 (5.7)	**3 (3.7)**	**4 (10.0)**	**0.0028**
Yes	37 (30.3)	**32 (39.0)**	**5 (12.5)**	
Not done	78 (63.9)	**47 (57.3)**	**31 (77.5)**	
**No. of fulfilled items from 8 items**				
0 item	2 (1.6)	**0**	**2 (5.0)**	** <0.0001**
1 item	5 (4.1)	**2 (2.4)**	**3 (7.5)**	
2 items	20 (16.4)	**3 (3.7)**	**17 (42.5)**	
3 items	28 (23.0)	**14 (17.1)**	**14 (35.0)**	
4 items	34 (27.9)	**30 (36.6)**	**4 (10.0)**	
5 items	26 (21.3)	**26 (31.7)**	**0**	
6 items	7 (5.7)	**7 (8.5)**	**0**	
Median (Q1,Q3)	4 (3, 5)	**4 (4, 5)**	**2 (2, 3)**	** <0.0001**
Min, max	0, 6	**1, 6**	**0, 4**	
Fulfill 3 regularly checked criteria: fever, splenomegaly, and cytopenias	61 (50.0)	**54 (65.9)**	**7 (17.5)**	** <0.0001**
**Early diagnostic tests for HLH^§^**				
Within 48 h	19 (15.6)	**17 (20.7)**	**2 (5.0)**	**0.0245**
Within 72 h	77 (63.1)	**63 (76.8)**	**14 (35.0)**	** <0.0001**
**Other tests within 48 h^¶^**				
TBil, >19 μmol/L	55 (45.1)	**45 (54.9)**	**10 (25.0)**	**0.0018**
ALT, >40 U/L	99 (81.1)	**75 (91.5)**	**24 (60.0)**	** <0.0001**
APTT, >48s	74 (60.7)	**62 (75.6)**	**12 (30.0)**	** <0.0001**
PT, >14s	85 (69.7)	**66 (80.5)**	**19 (47.5)**	**0.0002**
INR, >1.5	34 (27.9)	26 (31.7)	8 (20.0)	0.1758

*ALT, Alanine aminotransferase; APTT, activated partial thromboplastin time; HLH, haemophagocytic lymphohistiocytosis; INR, international normalized ratio; NK-cell, natural killer cell; PT, prothrombin time; TBil, total bilirubin*.

*Values in bold are statistically significant (p <0.05)*.

† *Results for soluble CD25 tests and genetic tests were not available within 72 h of admission*.

‡ *p-value not estimated because no negative results was detected*.

§ *Accomplishing all diagnostic tests from the HLH-2004 criteria except for NK-cell activity, soluble CD25, and genetic tests*.

¶ *Displayed according to local lab reference ranges*.

Table 4 displays factors associated with a hospital admission-to-diagnosis time lag > 3 days. Patient age <1 year was associated with a higher risk of admission-to-diagnosis > 3 days (OR = 3.08, 95%CI: 1.11, 8.57). Hospital admission through the PICU was negatively associated with admission-to-diagnosis > 3 days (OR = 0.38, 95%CI: 0.17, 0.86). EBV infection was also negatively associated with admission-to-diagnosis> 3 days (OR = 0.21, 95%CI: 0.09, 0.49). Diagnosis establishment without NK-cell activity and sCD25 tests, the presence of hypofibrinogenemia, and a ferritin level ≥ 500 μg/L were negatively associated with risk of admission-to-diagnosis > 3 days (all ORs <1, *p*-values <0.05). Several symptoms and laboratory findings within 48 h of hospital admission were associated with an earlier diagnosis, including fever, splenomegaly, and cytopenias, hypertriglyceridemia and/or hypofibrinogenemia, and haemophagocytosis, as well as elevated ferritin, ALT, TBil, APTT, PT, and INR. Diagnosis of sepsis within 48 h was also negatively associated with admission-to-diagnosis > 3 days (OR = 0.43, 95%CI: 0.2, 0.95). Performance of HLH diagnostic tests within 48 and 72 h of hospital admission was significantly associated with a lower risk of admission-to-diagnosis > 3 days (OR = 0.2 and OR = 0.16, respectively; both *p*-values <0.05).

**Table 4:** Factors associated with a late HLH diagnosis.

**Factor^†^**	**Hospital admission to diagnosis** **>3 days**	
**(l0ptr0pt)2-3**	**OR (95%CI)**	***P***
Age, <1 year	3.08 (1.11, 8.57)	0.0308
PICU admitted	0.38 (0.17, 0.86)	0.0202
EBV infection	0.21 (0.09, 0.49)	0.0002
**Fulfillment of HLH-2004 diagnostic criteria**		
Diagnosis established without NK-cell activity and soluble CD25 tests	0.32 (0.11, 0.94)	0.0381
Hypofibrinogenemia, ≤ 1.5 g/L	0.32 (0.12, 0.9)	0.0308
Ferritin ≥ 500 μg/L	0.18 (0.04, 0.73)	0.017
**Abnormal findings within 48 h after hospital admission**		
Fever, splenomegaly, and cytopenias	0.11 (0.04, 0.28)	<0.0001
Splenomegaly	0.33 (0.14, 0.75)	0.0086
Cytopenias	0.13 (0.06, 0.31)	<0.0001
Hemoglobin <90 g/L	0.21 (0.09, 0.47)	0.0002
Platelets <100 × 10^9^/L	0.17 (0.07, 0.42)	0.0001
Neutrophils <1.0 × 10^9^/L	0.33 (0.15, 0.72)	0.0055
Hypertriglyceridemia and/or hypofibrinogenemia	0.15 (0.06, 0.34)	<0.0001
Hypertriglyceridemia, ≥ 3.0 mmol/L	0.3 (0.12, 0.76)	0.0107
Hypofibrinogenemia, ≤ 1.5 g/L	0.25 (0.11, 0.56)	0.0007
Hemophagocytosis	0.07 (0.02, 0.33)	0.0006
Ferritin ≥ 500 μg/L	0.22 (0.08, 0.63)	0.0046
TBil, >19 μmol/L	0.27 (0.12, 0.63)	0.0025
ALT, >40 U/L	0.14 (0.05, 0.38)	0.0001
APTT, >48s	0.14 (0.06, 0.32)	<0.0001
PT, >14s	0.22 (0.1, 0.5)	0.0003
Sepsis	0.43 (0.2, 0.95)	0.0357
**Early diagnostic tests for HLH^‡^**		
Within 48 h	0.2 (0.04, 0.92)	0.0386
Within 72 h	0.16 (0.07, 0.37)	<0.0001

*ALT, alanine aminotransferase; APTT, activated partial thromboplastin time; CI, confidence interval; EBV, Epstein-Barr Virus; HLH, haemophagocytic lymphohistiocytosis; INR, international normalized ratio; NK-cell, natural killer cell; OR, odds ratio; PICU, pediatric intensive care unit; PT, prothrombin time; TBil, total bilirubin*.

† *Only presented factors with significant associations*.

‡ *Accomplishing all diagnostic tests from the HLH-2004 criteria except for NK-cell activity, soluble CD25, and genetic tests*.

### Sensitivity Analysis and Additional Analysis

Because admission through PICU and EBV infection were negatively associated with an admission-to-diagnosis time lag > 3 days, we compared the diagnostic characteristics between PICU-admitted and non-PICU-admitted patients (Supplementary Table 2), and between EBV-positive and non-EBV-positive patients (Supplementary Table 3). In our cohort, 78.8% of the PICU-admitted patients with HLH were diagnosed in ≤3 days and 58.6% of non-PICU-admitted patients were diagnosed in ≤ 3 days (*p* < 0.05, Supplementary Table 2). Furthermore, PICU-admitted patients had a higher rate of early screening for HLH (HLH tests within 72 h: 73.1%for PICU-admitted patients and 55.7% for non-PICU-admitted patients, *p* = 0.0493) and had a lower 30-day OS rate (58.8% for PICU-admitted patients and 76.5% for non-PICU-admitted patients, *p* = 0.0469). Patients with EBV infection had shorter symptom-to-diagnosis time lags and admission-to-diagnosis time lags (Supplementary Table 3). They also had a higher rate of HLH tests performed within 72 h (72.3% for EBV-positive patients and 43.6% for non-EBV-positive patients, *p* = 0.0022) and had a better 30-day OS rate (77.8% for EBV-positive patients and 50% for non-EBV-positive patients, *p* = 0.0031).

Tables 1, 4 showed that Age <1 year was associated with a higher risk of admission-to-diagnosis > 3 days, however, comparisons of diagnostic characteristics (days between first symptom to hospital admission, fulfillment of 3 regularly checked criteria, and rates of early diagnostic tests within 48 and 72 h) between <1 year and ≥ 1 year age groups showed non-significant results (*p* > 0.05).

Table 1 shows that the 30-day OS rates did not differ significantly among time lag categories. To determine whether this finding was affected by the cut-off value for time lag categorization, we compared 30-day OS rates between time lag categories using other cut-off points (e.g., admission-to-diagnosis time lag ≤ 4 days vs. > 4 days) (Supplementary Table 4). Similar non-significant associations were observed in these sensitivity analyses (Supplementary Table 4).

## Discussion

In this HLH cohort, the median lengths between first symptom onset to HLH diagnosis was 12 days (range 4–71 days); and the median lengths between hospital admission to HLH diagnosis was 2 days (range 0–23 days), and 67.2% patients were diagnosed within 3 days of admission. Age < 1 year was associated with a higher risk of admission-to-diagnosis time lag > 3 days. The following factors were negatively associated with admission-to-diagnosis > 3 days: Epstein–Barr virus infection; admission through pediatric intensive care unit; diagnosis established without NK-cell activity and soluble CD25 tests; the performance of all readily available diagnostic tests for HLH (within 48 and 72 h); concurrent fever, splenomegaly, and cytopenias within 48 h; hemophagocytosis, hypertriglyceridemia and/or hypofibrinogenemia within 48 h; and elevated ferritin, total bilirubin, alanine aminotransferase, and prothrombin time within 48 h.

Our findings show that the OS rates did not differ significantly among time lag categories, but these results do not imply that early diagnosis will not improve survival. Importantly, although treatment could affect OS, our study did not control for treatment, which interferes with comparison of OS rates among groups. For example, more patients in the admission-to-diagnosis > 3 days group received dexamethasone before confirmed diagnosis of HLH. Furthermore, patients within the different time lag groups had distinct baseline characteristics. Our data showed that patients diagnosed earlier (symptom-to-diagnosis ≤ 12 days) had a shorter symptom-to-admission interval, implying that the disease progressed quickly in at least some of the rapidly diagnosed patients. Notably, 50% of the patients diagnosed ≤ 3 days were admitted to the hospital directly to the PICU, but only 27.5% of the patients diagnosed >3 days were admitted directly through the PICU. Our previous study showed that PICU admission was a risk factor for early death in patients with HLH (10). Additionally, patients diagnosed ≤ 3 days showed higher rates of abnormal TBil, ALT, APTT, and PT within 48 h. They also had higher rates of sepsis diagnosed within 48 h. These findings indicate that patients with a shorter admission-to-diagnosis time lag were more likely to have severe illness at admission. Because rapid progression and deterioration are devastating in patients with HLH, timely diagnosis and treatment are crucial for early survival, especially for patients who already admitted to PICU (31–33); therefore, once suspected, adequate diagnostic tests (including NK-cell activity and sCD25 tests) for HLH should be arranged immediately for patients with severe illness and/or admitted to PICU.

Age <1 year was associated with a longer admission-to-diagnosis time lag. This association was less likely to be mediated by the difference in diagnostic procedures among different age groups, as comparisons of the days between first symptom to hospital admission, fulfillment of three regularly checked criteria, and the rates of early diagnostic tests within 48 and 72 h between < 1 year and ≥ 1 year age groups showed non-significant results. A possible explanation is that the disease progression pattern among different age groups was different and could have affected the HLH diagnostic time lag. The generalizability and the cause of this association worth further investigation, which could guide the improvement of diagnostic and management procedures among different age groups.

EBV is the most consistently reported viral infection associated with HLH, especially in Asian populations (8, 34–36). Given this clear association between EBV infection and HLH, EBV-positive patients in our hospital are closely monitored for potential HLH. Generally, patients with EBV infection and showed other HLH-like features would be screened for HLH more rapidly than non-EBV patients. Our results show that 72.3% of patients with EBV-related HLH had diagnostic tests within 72 h. Among patients without confirmed EBV infection, the early test rate was 43.6%. Furthermore, more patients with EBV-related HLH had concurrent fever, splenomegaly, and cytopenias within 48 h, which are signs for HLH susceptibility and might encourage early monitoring. Consequently, EBV infection was negatively associated with admission-to-diagnosis > 3 days. Further research is needed to investigate risk factors and early signals for HLH in patients with EBV infection, which will help identify patients with a high risk of EBV-HLH and enables more efficient diagnosis both in Asia and non-Asia populations.

The most frequently met HLH-2004 criteria were cytopenias (100%), hypertriglyceridemia and/or hypofibrinogenemia (100%), splenomegaly (91.8%), and fever (91.0%). These findings were similar to those in a previous study (11). Consistent with our hypothesis, patients with a later HLH diagnosis presented to the hospital with fewer HLH-like clinical characteristics. The median numbers of fulfilled diagnostic items within 48 h of admission were 4 (range 1–6) and 2 (range 0–4) among admission-to-diagnosis time lag ≤ 3 days and >3 days groups (*p* < 0.0001). Consequently, patients with more HLH-like features underwent diagnostic/monitoring tests sooner than did patients with fewer HLH-like features, in agreement with our findings that the performance of all early tests for HLH (within 48 and 72 h) was associated with earlier diagnosis. A prolonged time lag between hospital admission and diagnosis may result from insufficient testing, but also could be caused by disease progression in patients who initially present with mild symptoms that later progress to obvious HLH during hospitalization (37). In the current cohort, some of the diagnostic items were not checked at hospital admission. Therefore, we could not distinguish patients with inadequate tests from those whose disease progressed to HLH after admission. However, fever, splenomegaly, and cytopenias were checked in all patients at admission. Within 48 h of admission, 50% of the patients exhibited these three features concurrently and 34.4% of the patients developed these features later. Thus, 84.4% of the patients eventually met these three criteria during hospitalization. These findings demonstrate that both adequate diagnostic tests and HLH susceptibility monitoring are essential for timely diagnosis of HLH.

Both the laboratory results turnaround time and availability of diagnostic tests can affect the efficiency of diagnostic procedure. The current diagnose procedure in our study center took up to 3 days (not including NK-cell activity, sCD25, and genetic tests). Under this procedure, only 15.6% patients received HLH diagnostic test results within 48 h, and this rate increased to 63.1% within 72 h. Therefore, to establish a rapid diagnostic procedure for HLH, improvement in laboratory results turnaround time is needed in our center.

A difficulty in applying the current diagnostic criteria is that tests for NK-cell activity and sCD25 are typically not available in low-resource settings (18). In some hospitals, including ours, these tests are available but they are conducted by third-party providers, and several days are needed to complete the tests and obtain the results. The proportions of patients tested for NK-cell activity and sCD25 in our hospital were low (74.6 and 36.1% respectively), as neither test was performed in the absence of a clinical suspicion of HLH or other related diseases. Our analysis revealed that NK-cell activity and/or sCD25 tests to establish a diagnosis of HLH were needed more often in the admission-to-diagnosis time > 3 days group, in other words, patients diagnosed later were those in whom HLH was more difficult to establish. Although we were unable to determine how many patients would have been diagnosed earlier if HLH diagnostic tests had been conducted sooner, our results demonstrate that the regularly checked features and tests are insufficient for the early identification of HLH patients.

The difficulties in applying the current diagnostic criteria for the early identification and diagnosis of HLH have been fully addressed in previous studies and review articles (12, 18, 21). However, this is the first study to provide a quantitative evaluation of the current diagnostic time gap distribution and an assessment of the patient characteristics associated with early or late diagnoses. Our findings reveal the need for more research aimed at improving the diagnosis of HLH, especially with regard to the following: First, studies are needed to draw a better and clear strategy to identify suspicious patients to take diagnostic tests for HLH and to evaluate disease severity during diagnostic testing. The current diagnostic procedure in our center identified patients who have met at least three diagnostic criteria (usually fever, splenomegaly, and cytopenias) as suspicious patients, and established the HLH diagnosis in 67.2% of HLH patients within 3 days. This procedure could be further improved by introducing evidence-based diagnostic rules which can better identify patients who should undergo diagnostic tests for HLH and when these tests should be repeated/monitored. Second, the current diagnostic criteria should be improved, by introducing new diagnostic markers that reduce the number of tests and/or by improving the diagnostic sensitivity of existing tests. Candidate markers for HLH should be verified in terms of their ability to diagnose HLH more rapidly than the current diagnostic criteria. Third, because not all criteria are fulfilled at disease presentation, the dynamic pattern of the clinical parameters as an evaluation for disease progression should be investigated, and a monitoring plan should be developed to guide the monitoring of patients who have suspected HLH but do not fulfill the diagnostic criteria.

The main limitation of our study was that it was a retrospective study conducted at a single center. Because of the retrospective design, the monitoring plan were not standardized in this study. The ability to rapidly diagnose HLH requires experienced medical staffs and sufficient resources, including the ability to conduct genetic, NK-cell activity, and sCD25 testing. The distribution of the diagnostic time and the influencing factors may vary depending on the study setting. However, this is the first study to examine the distribution of HLH diagnostic times in HLH and the associated patient characteristics. Therefore, our results provide an important reference for future studies. Hunan Children's Hospital is one of the largest tertiary children's hospitals in central China and has treated hundreds of pediatric HLH patients. Thus, our findings are likely to be generalisable, especially for regions with a comparable setting and similar medical resources. The second limitation also derived from the retrospective design: failure to conduct all necessary diagnostic tests, especially NK-cell activity, sCD25, and genetic tests, may have led to some missed HLH diagnoses. Lachmann et al. retrospectively collected the medical records of adult intensive-care-unit patients with a serum ferritin level of ≥ 500 μg/L and available data for at least four HLH-2004 criteria, then retrospectively diagnosed those patients with HLH (32); they found that seven of nine patients with HLH were undiagnosed. Furthermore, patients who lived longer were more likely to have been accurately diagnosed. Assuming that the number of patients with undiagnosed HLH in the pediatric population is also not small, studies that investigate all eight HLH-2004 criteria among pediatric patients who died from unknown diseases and exhibited HLH-like features could improve our understanding of instances in which HLH is not detected. Besides, as only a few patients in this cohort had taken genetic test, the incidence of primary HLH could have been underestimated. The third limitation was that the date of symptom onset recorded in the medical records was reported by the patients' parents, which might have been subject to recall bias. Other patient parameters assessed before hospital admission were not available and were therefore excluded from analysis. Finally, this study included only patients with HLH, and therefore could not identify new factors that may assist in early diagnosis. Studies of hospital-based cohorts which includes both HLH and non-HLH patients will facilitate the development of a diagnostic algorithm to distinguish patients with HLH from all patients who present to the hospital.

To conclude, performance of adequate diagnostic tests is essential for early diagnosis of HLH. A shorter diagnostic time lag could be a sign of rapid deterioration. Once suspected, immediate and adequate diagnostic tests for HLH should be arranged for PICU patients. Improvements in diagnostic procedures and monitoring plans are needed to promote early diagnosis of HLH.

## Data Availability Statement

The raw data supporting the conclusions of this article will be made available by the authors, without undue reservation.

## Ethics Statement

The studies involving human participants were reviewed and approved by Medical Ethics Committee of the Hunan Children's Hospital. Written informed consent from the participants' legal guardian/next of kin was not required to participate in this study in accordance with the national legislation and the institutional requirements.

## Author Contributions

ZX, XLu, XLi, and HY designed this study. ZX, XLi, and HY wrote the manuscript. All authors participated in the data analysis, interpretation, and agree to be accountable for the content of the work.

## Conflict of Interest

The authors declare that the research was conducted in the absence of any commercial or financial relationships that could be construed as a potential conflict of interest.
