# Supplementary material for: Diagnostic Time Lag of Pediatric Haemophagocytic Lymphohistiocytosis and Patient Characteristics: A Retrospective Cohort Study
Source: Front Pediatr. 2021 Jun 17;9:692849. doi: 10.3389/fped.2021.692849 (PMC8247774; doi:10.3389/fped.2021.692849)
Supplement: Supplementary file 1 [file Table_1.DOCX]

**STable1.** Concomitant diagnosis within 48 hours of hospital admission, n(%)

| Test/ diagnosis | All | Hospital admission to diagnosis | | |
| --- | --- | --- | --- | --- |
|  |  | ≤3 days | > 3 days | *p* |
| Total | 122 | 82 | 40 |  |
| Sepsis | 80 (65.6) | **59 (72.0)** | **21 (52.5)** | **0.0338** |
| Malignancy | 2 ( 1.6) | 1 ( 1.2) | 1 ( 2.5) | 0.5501 |
| Autoimmune disorders | 2 ( 1.6) | 1 ( 1.2) | 1 ( 2.5) | 0.5501 |
| CNS disease | 11 ( 9.2) | 7 ( 8.6) | 4 (10.3) | 0.7467 |
| Pneumonia | 87 (71.3) | 58 (70.7) | 29 (72.5) | 0.8394 |
| Respiratory failure | 28 (23.0) | 18 (22.0) | 10 (25.0) | 0.7070 |
| Coagulopathy | 31 (25.4) | 21 (25.6) | 10 (25.0) | 0.9421 |
| Gastrointestinal disorder | 17 (13.9) | 8 ( 9.8) | 9 (22.5) | 0.0564 |
| Acute kidney injury | 5 ( 4.1) | 4 ( 4.9) | 1 ( 2.6) | 1 |
| Shock | 9 ( 7.4) | 7 ( 8.5) | 2 ( 5.0) | 0.7163 |
| Hepatic | 61 (50.0) | 41 (50.0) | 20 (50.0) | 1 |
| Myocardial damage | 38 (31.1) | 26 (31.7) | 12 (30.0) | 0.8484 |
| Heart failure | 28 (23.0) | 18 (22.0) | 10 (25.0) | 0.707 |

CNS, central nervous system disease.

Values in bold are statistically significant (*p*<0.05).

**STable2.** Diagnostic characteristics according to admission department

| Factor | Admission department, n(%) | | p |
| --- | --- | --- | --- |
|  | PICU  (n=52 ) | Other departments  (n=70) |  |
| First symptom to hospital admission (days) |  |  |  |
| Median (Q1,Q3) | 8 (6, 12) | 8 (6, 11) | 0.8638 |
| Min, max | 3, 38 | 2, 60 |  |
| First symptom to diagnosis |  |  |  |
| ≤12 days | 33 (64.7) | 34 (50.7) | 0.1389 |
| >12 days | 18 (35.3) | 33 (49.3) |  |
| Hospital admission to diagnosis |  |  |  |
| ≤3 days | **41 (78.8)** | **41 (58.6)** | **0.0183** |
| > 3 days | **11 (21.2)** | **29 (41.4)** |  |
| Fulfill 3 regularly checked criteria: fever, splenomegaly, and cytopenias | 44 (84.6) | 59 (84.3) | 0.9604 |
| Diagnosis established without NK-cell activity and soluble CD25 tests | 46 (88.5) | 60 (85.7) | 0.6566 |
| Fulfill 3 regularly checked criteria within 48h: fever, splenomegaly, and cytopenias | 30 (57.7) | 31 (44.3) | 0.1430 |
| Early diagnostic tests for HLH ^†^ |  |  |  |
| Within 48 hours | 9 (17.3) | 10 (14.3) | 0.6489 |
| Within 72 hours | **38 (73.1)** | **39 (55.7)** | **0.0493** |
| 30-day outcome |  |  |  |
| Survive | **30 (58.8)** | **52 (76.5)** | **0.0469** |
| Non-survive | 21 (41.2) | 16 (23.5) |  |

NK-cell, natural killer cell; PICU, paediatric intensive care unit.

Values in bold are statistically significant (*p*<0.05).

^†^Early diagnostic tests included all tests from the HLH-2004 criteria except for NK-cell activity and soluble CD25 tests.

**STable3.** Diagnostic characteristics according to EBV infection status

| Factor | EBV status, n(%) | | *p* |
| --- | --- | --- | --- |
|  | Positive  (n=83) | Negative/not done  (n=39) |  |
| First symptom to hospital admission (days) |  |  |  |
| Median (Q1,Q3) | 7 (6, 10) | 10 (6, 14) | 0.1635 |
| Min, max | 4, 60 | 2, 60 |  |
| First symptom to diagnosis |  |  |  |
| ≤12 days | **52 (65.8)** | **15 (38.5)** | **0.0059** |
| >12 days | **27 (34.2)** | **24 (61.5)** |  |
| Hospital admission to diagnosis |  |  |  |
| ≤3 days | **65 (78.3)** | **17 (43.6)** | **0.0001** |
| > 3 days | **18 (21.7)** | **22 (56.4)** |  |
| Fulfill 3 regularly checked criteria: fever, splenomegaly, and cytopenias | 72 (86.7) | 31 (79.5) | 0.3024 |
| Diagnosis established without NK-cell activity and soluble CD25 tests | 73 (88.0) | 33 (84.6) | 0.6107 |
| Fulfill 3 regularly checked criteria within 48h | **49 (59.0)** | **12 (30.8)** | **0.0036** |
| Early diagnostic tests for HLH^†^ |  |  |  |
| Within 48 hours | 16 (19.3) | 3 ( 7.7) | 0.0998 |
| Within 72 hours | **60 (72.3)** | **17 (43.6)** | **0.0022** |
| 30-day outcome |  |  |  |
| Survive | **63 (77.8)** | **19 (50.0)** | **0.0031** |
| Non-survive | **18 (22.2)** | **19 (50.0)** |  |

EBV, Epstein-Barr Virus; HLH, haemophagocytic lymphohistiocytosis; NK-cell, natural killer cell.

^†^Early diagnostic tests included all tests from the HLH-2004 criteria except for NK-cell activity and soluble CD25 tests.

**STable 4.** Sensitivity analysis for the 30-day outcome comparison using different cut-off points

| Cut-off days | 30-day overall survival, n(%) | | *p* |
| --- | --- | --- | --- |
|  | ≤ cut-off days | > cut-off days |  |
| **Admission-to-diagnosis time lag** |  |  |  |
| 2 days | 41 (68.3) | 41 (69.5) | 1 |
| 3 days | 52 (65.0) | 30 (76.9) | 0.2117 |
| 4 days | 57 (66.3) | 25 (75.8) | 0.3805 |
| 9 days | 67 (66.3) | 15 (83.3) | 0.1783 |
| 10 days | 71 (67.6) | 11 (78.6) | 0.5450 |
| 11 days | 73 (67.0) | 9 (90.0) | 0.1701 |
| **Symptom-to-diagnosis time lag** |  |  |  |
| 12 days | 36 (73.5) | 42 (63.6) | 0.3154 |
| 13 days | 33 (75.0) | 45 (63.4) | 0.2227 |
| 14 days | 32 (78.0) | 46 (62.2) | 0.0975 |
| 15 days | 28 (77.8) | 50 (63.3) | 0.1378 |
| 16 days | 25 (78.1) | 53 (63.9) | 0.1831 |
| 17 days | 23 (76.7) | 55 (64.7) | 0.2630 |
